# The Association Between Depression and Obesity Among Adults in Jeddah, Saudi Arabia, in 2022

**DOI:** 10.7759/cureus.35428

**Published:** 2023-02-24

**Authors:** Turki M Alkharji, Rwan S Alharbi, Emad A Bakhsh, Mohammed Alghalibi, Robba A Alraddadi

**Affiliations:** 1 Family Medicine, Ministry of Health (MoH), Jeddah, SAU; 2 Pediatrics, Ministry of Health (MoH), Jeddah, SAU

**Keywords:** saudi arabia, patient health questionnaire (phq-9), bmi, body mass index, overweight

## Abstract

Background

Depression has emerged as a significant contributor to the worldwide loss of disability-adjusted life years. Simultaneously, obesity is regarded as a substantial global health issue. The co-existence of depression and obesity can further exacerbate negative health outcomes.

Objective

The objective of this study was to investigate the relationship between depression and obesity in adult populations in Jeddah, Saudi Arabia. Further, the study aimed to examine the impact of confounding variables and their association with depression and obesity.

Methods

This analytical cross-sectional study utilized an interviewer-assisted questionnaire to collect data from adult participants aged 18 y/o or older attending primary healthcare centers at the Ministry of Health in Jeddah. The study was conducted at primary healthcare centers in Jeddah city, Saudi Arabia. The questionnaire included information on demographic characteristics, comorbidities, weight and height, and the Patient Health Questionnaire-9 (PHQ-9) tool for assessing the incidence of depression.

Results

A total of 397 individuals were included in the study with more than 50% of the participants between 26 and 45 years. The majority of the participants were males in the study (56.9%). The self-reported chronic diseases by the participants included diabetes mellites (25.9%), hypertension (23.7%), and dyslipidemia (19.9%). The study found that 12.8% of respondents had depression, 11.1% had anxiety, and 2.5% had obsessive-compulsive disorder. A total of 29.7% of participants had a PHQ-9 score of 10 or more. A significant negative linear correlation was found between the PHQ-9 score of the participants and their body mass index (BMI) results. However, this association did not remain significant when the chi-square test was used. Moreover, diabetes mellites and hypertension among the study sample were significantly associated with moderate to severe depression (p-values = .006 and = .005, respectively). The PHQ-9 score was negatively correlated with the participants' BMI, with a coefficient of -.190 (p-value < .001).

Conclusion

In the current study, the majority of obese participants displayed symptoms of depression ranging from mild to moderate. However, no significant correlation was established between depression and BMI.

## Introduction

Overweight and obesity are medical conditions that point to an excessive buildup of body fat and have detrimental effects on one's health. Obesity and overweight have been recognized as risk factors for a number of diseases, including diabetes, different cancers, cardiovascular disorders, and hypertension by epidemiological research. The health of people is at risk in many regions owing to the rising incidence of high body mass index (BMI) and the mortality that follows. It also has detrimental impacts on health and puts a financial burden on individuals and society [1].

Obesity has raised a major global public health issue [2]. In both developed countries and developing countries, the incidence of obesity has dramatically increased over the last several decades [3], approximately doubling between 1980 and 2008 [4,5]. The rate of adult obesity in the Middle East, which encompasses parts of North Africa and western Asia, is alarmingly high (24.5%), matching those from other European nations like the United Kingdom (22.9%) and Germany (26.3%). Also in Middle Eastern countries, in particular, obesity has been far more prevalent among women (30.6%) than men (16.6%). The primary causes of the high incidence of obesity and its comorbidities in the Middle East have been recognized as the major behavioral transformation highlighted by the increasingly sedentary lifestyle, the replacement of regular diet with a westernized food pattern, and the lack of exercise as well as other social and cultural factors [6].

A recent study undertaken in nine Middle Eastern countries established a correlation between the region's rising non-communicable disease occurrence and the rise in obesity, including diabetes, cardiovascular disease, and cancer [7]. Obesity may have a significant psychological burden in addition to exerting a negative impact on physical health [8]. In fact, studies on the general population in Western nations have focused more and more on the relationship between obesity and depression. In the general adult population, obesity has a strong positive association with depression according to a recent meta-analytic assessment of cross-sectional community-based research [9]. Furthermore, women have been more strongly associated with this relationship than men [9]. According to findings from another statistical analysis of 15 cross-sectional studies, depression in the general public is positively associated with abdominal obesity [10]. Additionally, a recent systematic review and meta-analysis of 15 longitudinal studies revealed a causal relationship between depression and obesity [11]. Since studies from Western populations are the only ones used to draw these conclusions on the link between fat and depression. It is important to look into the connection between these two variables in these communities considering the potential psychological burden caused by the rising obesity rates among adults in the Middle East. Health policymakers must prioritize interventions to prevent and control weight gain and obesity based on the most recent data on the prevalence and trends of these conditions [12].

As established in the literature, being overweight and obese are substantial issues in Saudi Arabia. Moreover, these disorders are associated with multiple health problems such as depression, which may decrease the individual quality of life significantly. Thus, this study aims to investigate the relationship between probable associated factors and depression among adults in Jeddah, Saudi Arabia.

## Materials and methods

This is an analytical cross-sectional study that utilized an interviewer-assisted questionnaire to collect data from participants. The study will be carried out at primary healthcare centers (PHCCs), belonging to the Ministry of Health in Jeddah city, which is the largest city located in the western region of the Kingdom of Saudi Arabia.

Adults residing in Jeddah city in the Kingdom of Saudi Arabia at the time of the study were invited. The sample size was estimated at 385 using a web-based sample size calculator (www.raosoft.com), with a 5% margin of error, 95% confidence level, and 50% response distribution. However, a total of 397 respondents were included in this study to overcome missing values.

Data collection technique and questionnaire

This study used a convenient sampling technique to include individuals from the target population attending PHCCs in Jeddah city, and the technique was the most appropriate in the settings to include a representative sample in number. The questionnaire was administered by the trained data collectors who are administrative workers at the PHCCs where they interviewed the study participants. The inclusion criteria included adults aged 18 y/o or older who attend PHCCs belonging to the Ministry of Health (MOH) in Jeddah city. The validated questionnaire was adapted from a previous study that was conducted in similar circumstances and included a comparative population to the current study target population [13]. The questionnaire included the following variables: demographic characteristics, comorbidities, and the Patient Health Questionnaire-9 (PHQ-9) tool. In addition, participants' weight and height were measured at the PHCCs to calculate BMI measures. The PHQ-9 tool is a validated tool to screen for depression, which is composed of nine items. The tool score ranges from 0 to 27, and based on the patient's score, they are labeled from mild to severe depression. A cut-off score of 10 or more is sensitive and specific for moderate to severe depression.

Statistical analysis

Data were analyzed using the Statistical Package for the Social Sciences (SPSS, version 29.0; IBM Corp., Armonk, NY). Proportions and frequencies were used to summarize the data. Moreover, inferential statistics using the chi-square test (χ^2^), the extension of the Fisher-Freeman-Halton exact test, and Spearman's correlation test were applied, and p-values < .05 were considered statistically significant.

Ethical consideration

Ethical approval was obtained from the research ethics committee of the Research and Studies department at the Directorate of Health Affairs in Jeddah city before data collection. A statement explaining the nature and the purpose of the study was included to gain the participants' consent before filling out the questionnaire. Participating in the study was voluntary and did not affect the care participants received. All data were handled anonymously, securely saved, and used for research purposes only.

## Results

A total of 397 individuals met the inclusion criteria and were included in the study analysis. More than 50% of the participants were between the ages of 26 and 45 years. Male respondents represent slightly more than half of the total study sample (56.9%), and 91.9% of the total number were Saudi residents. Furthermore, 45.3% hold a bachelor's degree, while 17.1% of the included participants have pursued higher education degrees. Detailed demographic data such as marital status and socioeconomic status are illustrated in Table 1.

**Table 1:** Demographic data SAR: Saudi riyal.

n = 397		N	%
Age (y/o)	18-25	67	16.9%
	26-35	90	22.7%
	36-45	117	29.5%
	46-55	63	15.9%
	56-65	47	11.8%
	>65	13	3.3%
Gender	Male	226	56.9%
	Female	171	43.1%
Nationality	Saudi	365	91.9%
	Non-Saudi	32	8.1%
Marital status	Single	105	26.4%
	Married	268	67.5%
	Divorced	18	4.5%
	Widowed	6	1.5%
Number of family dependents	1	74	18.6%
	2	49	12.3%
	3	68	17.1%
	>3	206	51.9%
Family income (SAR)	<5000	78	19.6%
	5000-10,000	101	25.4%
	10,001-15,000	79	19.9%
	>15,000	139	35.0%
Educational level	No official education	2	0.5%
	Primary school	6	1.5%
	Secondary school	23	5.8%
	High school	118	29.7%
	Bachelor	180	45.3%
	Higher education	68	17.1%

The study participants self-reported any chronic diseases they were living with at the time of the study, and diabetes mellites (25.9%), hypertension (23.7%), and dyslipidemia (19.9%) were the most prevalent chronic diseases among the study participants. Furthermore, 12.8% of the respondents reported an established diagnosis of depression, anxiety (11.1%), and obstructive-compulsive disorder (2.5%). In addition, the study investigator measured the participants' weight and height to attain their BMI, and the results show that 65.5% were obese, and 17.6% were overweight. The study screened participating individuals for depression using PHQ-9, and 29.7% had a total score of 10 points or more (Table 2).

**Table 2:** Comorbidities PHQ-9: Patient Health Questionnaire-9; BMI: Body mass index; DM: Diabetes mellites; HTN: Hypertension; OCD: Obsessive-compulsive disorder.

n = 397		N	%
PHQ-9	No depression to mild depression	279	70.3%
	Moderate to severe depression (≥10)	118	29.7%
BMI	Underweight	5	1.3%
	Healthy weight	62	15.6%
	Overweight	70	17.6%
	Obesity	260	65.5%
Use of medications		156	39.3%
DM		103	25.9%
HTN		94	23.7%
Dyslipidemia		79	19.9%
Thyroid disorders		17	4.3%
Depression		51	12.8%
Anxiety		44	11.1%
OCD		10	2.5%
Bipolar		7	1.8%
Other psychiatric disorders		8	2.0%

The current study investigated all demographic variables such as age, gender, marital status, educational level, and family income for the possible association with moderate to severe depression based on the PHQ-9 score. However, none of the demographic factors showed a statistically significant association with moderate to severe depression. Meanwhile, patients with suggested moderate to severe depression were more likely to use medication at the time of data collection (p-value < .001). Moreover, diabetes mellites among the study sample was associated with moderate to severe depression, and this association achieved statistical significance (p-value = .006). In addition, 33.1% of participants with suggestive moderate to severe depression were diagnosed with hypertension, and this association was statistically significant (p-value = .005) as shown in Table 3. Moreover, the PHQ-9 score of the study participants was negatively correlated with their BMI results with a coefficient of .190 (p-value < .001).

**Table 3:** Associated factors with moderate to severe depression The Chi-square test and *Fisher’s exact test are used. PHQ-9: Patient Health Questionnaire-9; BMI: Body mass index; DM: Diabetes mellites; HTN: Hypertension; SAR: Saudi riyal.

n = 397		PHQ-9				
		No depression to mild depression		Moderate to severe depression (≥10)		
		N	%	N	%	p-values
Gender	Male	165	59.1%	61	51.7%	.184
	Female	114	40.9%	57	48.3%	
Age	18-25	42	15.1%	25	21.2%	.179*
	26-35	65	23.3%	25	21.2%	
	36-45	81	29.0%	36	30.5%	
	46-55	45	16.1%	18	15.3%	
	56-65	33	11.8%	14	11.9%	
	>65	13	4.7%	0	0.0%	
Income (SAR)	<5000	51	18.3%	27	22.9%	.588
	5000-10,000	70	25.1%	31	26.3%	
	10,001-15,000	55	19.7%	24	20.3%	
	>15,000	103	36.9%	36	30.5%	
BMI	Underweight	3	1.1%	2	1.7%	.806*
	Healthy weight	45	16.1%	17	14.4%	
	Overweight	47	16.8%	23	19.5%	
	Obesity	184	65.9%	76	64.4%	
Use of medications	No	188	67.4%	53	44.9%	
	Yes	91	32.6%	65	55.1%	
DM	No	218	78.1%	76	64.4%	.006
	Yes	61	21.9%	42	35.6%	
HTN	No	224	80.3%	79	66.9%	.005
	Yes	55	19.7%	39	33.1%	

## Discussion

The co-occurrence of depression and obesity is common due to intersecting pathophysiology and shared biological pathways, often leading to negative health implications [14,15]. In the current study, we observed a high rate of obese and overweight participants in this study. Almost two-thirds of the participants were obese, which was a much higher percentage compared to previous studies [13,16,17]. Al-Rethaiaa et al., in their cross-sectional study, reported that 21.8% of university students were overweight, and 15.7% were obese [16]. In another study from Saudi Arabia, Alshahrani et al. reported a prevalence of 38.4% obese individuals, whereas 44.2% were overweight [17]. Our findings were much higher compared to a study from an Eastern province in Saudi Arabia [13]. Almarhoon et al. reported that 30.5% of participants in their study met the criteria of overweight and 26.4% of obese [13]. A national survey of 4709 participants from 13 regions in Saudi Arabia in 2020 reported a national prevalence of 24.7% [18].

In the current study, we found a significant negative linear correlation between the PHQ-9 score of the participants and their BMI results. However, the association using the chi-square test was not significant. According to our PHQ-9 results, almost one-third of participants (29.7%) had moderate to severe depression. The PHQ-9 has been extensively researched and has been found to be the most precise tool for the assessment of depression [19-21]. The PHQ-9 is a validated tool that is recommended to be incorporated as a fundamental aspect of a comprehensive screening methodology in a two-stage screening process for depression [22]. This was in line with Almarhoon et al. who observed moderate to severe depression in 34.8% of their study participants [13]. However, our findings were much higher compared to those of Al-Qadhi et al., who reported that only 1% of participants in their study had severe depression, whereas 13.4% and 4.4% had moderate and moderate-severe prevalence, respectively [23]. In our study, most cases of depression were seen among overweight and obese individuals (19.5% and 41.7%), respectively. Carey et al., in their investigations of obese participants from 12 Australian general practices, reported much lower percentages compared to our study [24]. They revealed that among overweight participants, 12% had depression, whereas 23% of depression was seen in obese participants [24]. A cross-sectional study by Garg et al. from India reported that 12% of participants had moderate depression, whereas 54% had mild depression [25]. Our results were supported by a cross-sectional study from Abha, Saudi Arabia, which found that 42.7% of participants had moderate to severe depression [26]. They also had a similar percentage of obese individuals (71%) in their study, which could explain the similarity in results [26].

In the current study, we observed a statistical significance between diabetes mellites, hypertension, and moderate to severe depression. These findings were supported by Stecker et al., who found a significant association between diabetes, obesity, depression, and hypertension (p < .05) [27]. We also found that a significantly higher number of participants were taking medications regularly at the time of the study (p < .001). Similar findings have been shared previously by Almarhoon et al., who reported that 18.3% of participants were regularly taking any medications during their study period [13]. This phenomenon can be attributed to the adverse metabolic effects associated with the administration of antidepressant medications [28]. Our finding showed that a significant proportion of participants exhibiting moderate to severe levels of depression were found to have a BMI of 30 or greater. This can guide further future interventions to aim at mitigating obesity in an attempt to improve mental health.

Although the current study found a disproportional distribution of depression between individuals in different BMI categories, this variation did not achieve statistical significance, which may be attributed to the limitation of the sample size of the current study. Therefore, the authors of the present study suggest further research with the inclusion of a larger sample size that effectively represents the normal distribution of BMI in the general population. Moreover, a nationwide multicenter study is suggested as it may reveal more precise and informed recommendations.

## Conclusions

The co-occurrence of depression and obesity has emerged as a major public health concern. Currently, the awareness level is rudimentary concerning this topic among the general population. In light of this, the present study was undertaken to explore the relationship between depression and obesity in Saudi Arabia. We found a significant association between moderate to severe depression, hypertension, and diabetes mellitus. Our findings were in line with previous studies on this subject. However, we reported a much higher prevalence of obese participants in the current study. Although the majority of obese participants were depressed, the study did not find a statistical significance between BMI and depression. The findings of the current study indicate the urgency for public health initiatives focused on enhancing consciousness among the susceptible population.
